# Preflight Contingency Planning Approach for Fixed Wing UAVs with Engine Failure in the Presence of Winds

**DOI:** 10.3390/s19020227

**Published:** 2019-01-09

**Authors:** Bulent Ayhan, Chiman Kwan, Bence Budavari, Jude Larkin, David Gribben

**Affiliations:** Signal Processing Inc., Rockville, MD 20850, USA; bulent.ayhan@signalpro.net (B.A.); bencebudavari@gmail.com (B.B.); judelarkin93@gmail.com (J.L.); david.gribben00@gmail.com (D.G.)

**Keywords:** contingency planning, automated landing, forced landing, path generation, wind forecast, UAV

## Abstract

Preflight contingency planning that utilizes wind forecast in path planning can be highly beneficial to unmanned aerial vehicles (UAV) operators in preventing a possible mishap of the UAV. This especially becomes more important if the UAV has an engine failure resulting in the loss of all its thrust. Wind becomes a significant factor in determining reachability of the emergency landing site in a contingency like this. The preflight contingency plans can guide the UAV operators about how to glide the aircraft to the designated emergency landing site to make a safe landing. The need for a preflight or in-flight contingency plan is even more obvious in the case of a communication loss between the UAV operator and UAV since the UAV will then need to make the forced landing autonomously without the operator. In this paper, we introduce a preflight contingency planning approach that automates the forced landing path generation process for UAVs with engine failure. The contingency path generation aims true reachability to the emergency landing site by including the final approach part of the path in forecast wind conditions. In the contingency path generation, no-fly zones that could be in the area are accounted for and the contingency flight paths do not pass through them. If no plans can be found that fulfill reachability in the presence of no-fly zones, only then, as a last resort, the no-fly zone avoidance rule is relaxed. The contingency path generation utilizes hourly forecast wind data from National Oceanic and Atmospheric Administration for the geographical area of interest and time of the flight. Different from past works, we use trochoidal paths instead of Dubins curves and incorporate wind as a parameter in the contingency path design.

## 1. Introduction

The use of unmanned aerial vehicles (UAVs) in the military and industry today is becoming more widespread, making airspace traffic even more crowded than before. The mishap rates in UAVs are several orders of magnitude greater than for manned aviation [1]. Considering these high mishap rates, it is understandable why the U.S. Department of Transportation’s Federal Aviation Administration (FAA) has initiated several programs and partnerships to increase the safety and reliable operation of UAVs [2]. To reduce mishap rates, the reliability of UAVs can be improved by using durable engines and communication equipment, strong structural materials, advanced condition-based maintenance and structural health monitoring procedures [3,4,5,6], robust fault diagnosis and prognosis algorithms [7,8,9], and robust [10,11,12,13,14,15,16] and fault tolerant controllers [17,18,19]. Another area to reduce mishap rates is well devised contingency plans which will take place in the aftermath of an emergency either to help UAV operators glide the UAV or make the UAV autonomously land itself at a crashing/ditching site or local airport runway if there is no reliable communication link available. In the case of an emergency due to full loss of thrust, the wind plays a critical role with respect to reachability of the emergency landing site [20]. Upon full loss of thrust due to engine failure, because of the wind impact, the UAV may not reach the designated landing site and crash into populated areas causing loss of lives. Thus, the wind impact on reachability needs to be addressed in path planning for engine loss contingencies. It is also important that in the event of an emergency that might happen at high altitudes, the UAV should choose a forced landing path that does not violate no-fly zones or stormy weather air zones not to further complicate the situation.

In light of the above statements, it would be fair to state that the contingency plans need to address the problem of how the fixed-wing UAV with full loss of thrust can be guided to land at an emergency landing site without violating no-fly zones while accounting for wind conditions. Before landing, the UAV must be aligned with the landing site orientation for final approach at a certain gliding airspeed, altitude and heading to meet true reachability conditions. By true reachability, we mean the UAV being able to make a touchdown through a final approach path rather than simply arriving over the landing site with some excessive altitude. There is an emergent need for automated contingency planning methods to support the UAV operators to safely glide the mishap aircraft and for autonomous emergency landing methods.

There are several past works that propose automated forced landing approaches in the event of full loss of thrust. In [21], a real-time path planning method which uses Dubins curves is introduced. The authors extend Dubins curves to 3D which consider aircraft dynamics and contain wind information in the guidance logic assuming that the wind velocities can be estimated by onboard instruments. In their work, the authors state that the amount of loss in altitude factored into the path planning equations does not fully consider the associated loss in altitude due to varying airspeeds and other atmospheric effects. In their path planning, from the point of emergency to the approach point, they assign two altitudes. The path angle is computed between the two altitudes. If the difference in altitude between the start and end positions results in a path angle that exceeds the maximum allowable path angle, another suboptimal path is selected to lose the approximate amount of altitude required. It is mentioned that their algorithm can generate the required number of helix spirals to lose the excess altitude before connecting the spirals with a Dubins path. Their path planning does not consider obstacles in the flight path. Because they observed vertical track error when the aircraft was following a helix spiral, as future work, they mentioned that they would experiment different techniques. Luis et al. [22] presented flight test results of forced landings involving an Unmanned Aircraft System (UAS), in a controlled environment. The path planning and guidance algorithms used in these flight tests were taken from [21].

In [23], a method for on-board computation of energy-optimized flight-paths for engine loss emergencies is proposed. Optimal flight paths are generated via a search graph through discretization and it is accounted for obstacles with different shape and position. The authors state that the altitude at the final approach point is ignored in the optimal path design. They only aim to match the desired coordinates for the approach point, desired heading angle and speed values while maximizing the altitude at the approach point. The authors assume that the pilot will be able to get rid of the excess altitude once near the landing site and they do not provide a full reachability analysis.

In [24], a reachability analysis method in the presence of a known wind profile is introduced. A maximum glide range is defined using gliding equations and separating the glide into turning phase and straight level glide descent. Since the approach and safe landing phases are not accounted for in the maximum glide range computation, the authors use a descent circuit based on the high-key low-key technique generally used by human pilots to calculate the true reachability of a landing site. The authors mention about the need to develop a more representative descent path to follow since they consider the high-key low-key technique as wasteful one and being more appropriate for human pilots but not for UAVs.

In [25], a Dubins path-based framework is introduced to find emergency landing paths for fixed wing airplanes in case of total loss of thrust. The shortest path using Dubins Curve is found under best glide assumption and the optimal final approach length is estimated by solving nonlinear equations.

In [26], the shortest path to the identified emergency landing is found using Dubins curves. Their method calculates the altitude drop for gliding along the planned shortest path. Using this altitude drop, it computes the UAV altitude over the landing point. If this has a negative value, it is decided that the aircraft could not reach it and this landing site is excluded. If the altitude over the landing point has a positive value, the aircraft has excessive altitude and the length of the final approach path is extended till there is no excessive altitude. The wind effects are not considered in the path planning and it is considered that there are no obstacles or no-fly zones along the planned path. The authors bring into readers’ attention that there is a risk that an aircraft cannot reach the landing site in windy weather condition even though their method confirms that the landing site is reachable.

In [27], an in-flight automatic contingency generator (ACG) for UAVs is discussed which avoids no-fly zones by constructing tangent vectors around the no-fly zones. The energy state of the UAV is used to determine the UAV’s glide range and to identify candidate landing site locations within the glide range. It is mentioned that forecast and actual wind data can be used to dynamically adapt the routes for wind effects on the turn radius and climb/descent performance capabilities.

In [28], an in-flight method for autonomous safe emergency landing of a powered UAV in the event of an engine failure is introduced. The method generates a landing approach trajectory including a downwind leg, an upwind leg terminating at a selected touchdown point, and a U-turn leg joining between the downwind leg and the upwind leg. The UAV is directed to the initiation point to follow the downwind leg. A glide ratio of the UAV is repeatedly determined based on current flight conditions. The glide error is computed continuously from actual sensor on-board measurements such as altitude lost, airspeed and measured wind. The method considers waiting paths to lose excessive altitude path. For this, the trombone-shape flight approach path is gradually extended in length on both sides and looked for convergence on the landing site location to meet reachability with the measured glide ratio. The method does not mention about no-fly zones in the vicinity of the targeted emergency landing site.

A risk-aware path planning strategy for UAVs in urban environments is proposed to compute a path that minimizes the risk to the population in [29]. A risk map is utilized for quantification of the risk which associates discretized locations of the space with a suitable risk cost. The proposed approach consists of two phases, off-line and on-line path planning. The off-line path planning searches for a globally optimal path considering the risk-map as a static environment with an A*-based algorithm called riskA* which uses a cost function that considers both path length and risk-cost. For on-line path planning, an algorithm called Borderland is used that identifies and adjusts only the portion of path involved by the inception of relevant dynamical changes in the risk factor. After the path planning procedure, to achieve a more suitable and realistic path, the authors use a smoothing procedure which involves Dubins curves. The path planning is for UAVs with functional engines and wind effects are not accounted for in the path planning.

A path re-planning problem to land a UAV under four types of critical situation is studied in [30] to minimize damages during an emergency landing. Three methods were proposed by the authors: Greedy Heuristic (GH), Genetic Algorithms (GA) and Multi-Population Genetic Algorithm (MPGA). It is reported that the methods are better dealing with battery problem and worse for landing the UAV under engine failure. The authors mention that this issue must be considered as future work to improve these methods for engine failure case. According to their simulation results with a flight simulator, in regards to reachability, in no wind condition, the UAV is found to deviate from the original route on average 8.65 m and the average error is found to reach around 92.33 m under winds of 40 knots. It is our interpretation that the authors must have considered using aircraft controls to minimize the wind effects during the flight instead of utilizing wind directly as a parameter in the path planning as we did. Related with that no discussions were found about how the excessive altitude is lost by the UAV through these algorithms and about the effect of air density variation on reachability as the UAV descends.

The maneuverability limitations of the aircraft is critical in path planning especially in emergency landings. In [31], design and simulation of an on-line algorithm which estimates the safe maneuvering envelope of aircraft is discussed. From the trim envelope, authors indicate that the maneuverability limitations of the aircraft through an optimal control formulation can be obtained by a robust reachability analysis.

In [32], path planning problem for emergency landing with fixed-wing aircraft experiencing the total loss of thrust is studied. The author proposes a novel RRT*-based algorithm for planning safe gliding emergency landing trajectories. The proposed algorithm generates a map of the required altitude allowing a safe gliding emergency landing as its byproduct. Dubins maneuvers are used in the path planning. The effects of wind in path planning do not seem to be considered in this work since no related discussion about wind can be found. The author doesn’t provide any mechanisms about how the excessive altitude is lost and about the final landing trajectory. It is stated that the excessive altitude is considered to be safe and the excess altitude of the aircraft can be left up to the pilot or can be solved during retrieving the final landing trajectory.

In [33], the integration of an Emergency Landing Planner (ELP) into the cockpit of a 6 DOF full-motion simulator is discussed. ELP is developed for manned aircrafts and is designed to assist pilots in choosing the best emergency landing site when damage or failures occur in an aircraft. It takes various factors into consideration such as the actual control envelope of the aircraft, distance to the site, weather along the route, characteristics of the approach path and the runway or landing site, and emergency facilities at the site. With respect to path planning, the term roadmap is defined as the topological representation of the environment that captures the connectivity of the free space. In ELP, roadmaps are generated by starting with a 2D visibility graph first, and augmenting the edge set in the vertical dimension to allow paths above, below or through obstacles. A hybrid discrete/continuous version of A* that searches for paths of low risk in this roadmap is then used in the path planning. At the end, ELP proposes possible routes and landing sites to the pilot, ranking them according to estimated risk.

In this work, we propose a preflight contingency planning approach that automatically generates flight paths for UAVs with full loss of thrust to land them at an emergency landing site without crossing over any no-fly zones in the area and while also accounting for varying wind conditions in the path design using hourly wind forecast data. It aligns the UAV with the landing site’s orientation for the final approach path at a proper heading angle in forecast wind. Loitering paths are designed near the identified emergency landing site to lose excessive altitude to have a proper altitude for final approach, and to meet true reachability.

We assume a constant glide speed throughout the contingency flight path. Air density values that vary according to altitude are accounted for in the sink rate computations of the aircraft. The sink rates during straight level gliding and turn are estimated separately and incorporated into the path design. The preliminary path that does not violate any no-fly zones is segmented and the minimum-time paths are designed using six different extremal types for each segment that are based on trochoidal curves. These six extremals are LSL, RSR, RSL, LSR, RLR and LRL, where L corresponds to a left turn, R corresponds to a right turn and S corresponds to travelling straight [34].

The wind forecast data vary both spatially and in altitude. We assume that in smaller regions, the variation in wind is negligible. This assumption allows us to design the path trajectory for a segment using the six extremals that require steady wind conditions. The steady wind assumption is only for a single segment and the wind data in the consecutive segments would be different. The partitioning of the path into shorter segments, thus, allows incorporating varying wind into the contingency path design. We assume that the UAV has some degree of flight control to adjust its heading while straight gliding or making a turn according to the identified extremal. Related with that we also assume that the transition to turning flight occurs instantaneously instead of accounting for the time that it would take to acquire the target bank angle and turn rate.

Our approach differs from the past works by being an off-line preflight contingency planning tool to support UAV operators whereas most of the past works focus on in-flight path planning for forced landing in the case of full loss of thrust. We envision our preflight contingency plans being uploaded to the UAVs and in the event of an emergency, these plans would then support the UAV operator for guiding the forced landing of the UAV. Or, in the event of a lost communication link, the UAV would autonomously land itself through minor modifications of the preflight contingency plans using its onboard sensors on the fly. Because it is an off-line approach, computation time constraints are not strict. In-flight path planning methods use onboard sensors to measure wind speed and direction and use continuous guidance and control adjustments to compensate for wind effects to maintain reachability to the designated landing site. Our approach utilizes hourly wind forecast data to incorporate wind effects to the contingency path design before the flight. Even though the downward wind forecasts are generally small values, our approach incorporates them in the altitude drop estimations.

Most of the past works utilize Dubins curves for path planning and assume that the UAV will have continuous guidance controls to compensate for the wind effects while tracking the Dubins-curve based trajectories. One of the contributions of our work is incorporating wind in the contingency path planning with the use of six different extremals based on trochoidal curves. According to [35], using trochoidal curves in path planning instead of Dubins curves is found to reduce the tracking error in actual UAV flight experiments which shows the importance of using trochoidal curves over Dubins curves in the presence of wind.

Another contribution is the incorporation of loitering paths in the path design if there is any excessive altitude to lose before the final approach. For marginal excessive altitude loss, our method systematically computes a time-constrained loitering path unlike [28] which uses a trombone shape and extends this path continuously to converge on the targeted emergency landing site with the continuously measured glide ratio values. In our method, the sink rates to compute the glide ratio are derived from mathematical equations accounting for atmospheric density variations rather than using measurement data like [28] which requires some learning. A novel time-constrained path design technique [36] is adapted which provides LRL and RLR type extremal trajectory design in the time-constrained path. In addition to time-constrained path design, extremal trajectory look-up tables are formed with a range of turn rates for different wind amplitude and directions. If the time-constrained path design does not provide a solution, the extremal look-up table is searched to determine a loitering path of which its travel time is closest to the required air time to lose the excessive altitude.

Section 2 provides the problem description. A detailed discussion is given in this section regarding path generation for preflight contingency planning given a primary flight path (PFP). Section 3 provides the detailed introduction of our proposed approach and the technical steps. The tools used for loitering path design to lose excessive altitude are discussed in this section as well. Section 4 provides the results and discussions for simulated scenarios and using flight data from an actual incident. Section 5 provides the conclusions and discussion of future work.

## 2. Problem Description

**Figure 1:**
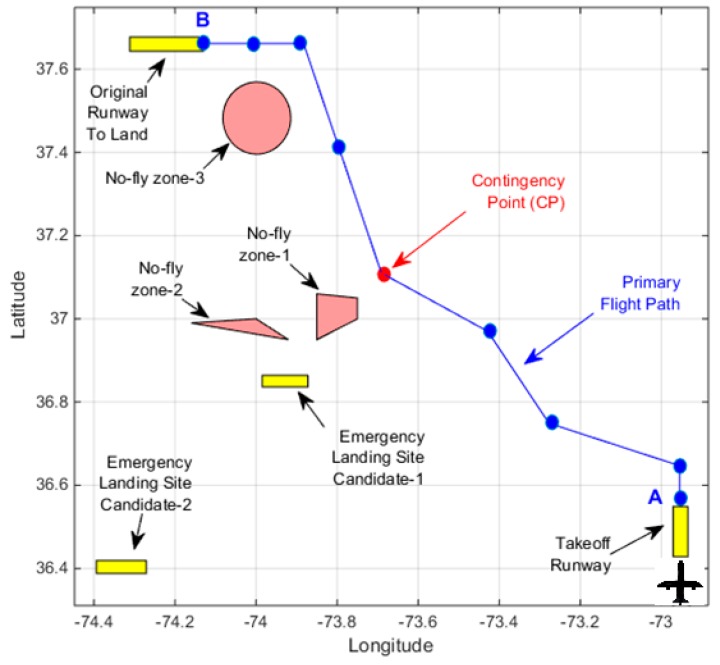
Illustration of the problem description.

Figure 1 illustrates the problem description. Suppose a UAV flight mission is planned in which the takeoff will take place at point *A* at time *t_A_* and the UAV is planned to land at point *B* at *t_B_*. A wind forecast is available for the area that covers the time duration in which the mission would take place. The mission primary flight path is known and depicted with blue line in Figure 1. The primary flight path consists of several waypoints and these waypoints are denoted by blue circles in Figure 1. Other than the primary flight path waypoints, UAV specific information such as the take-off weight, travel time from one waypoint to another, and estimated fuel consumptions at each waypoint along the flight path, etc., are available. The term theater is used in this work for the geographical area in which the primary flight path takes place. Within this theater, there could be several no-fly zones which the UAV should not cross through during its flight. Contingency plans are needed before the flight mission takes place to respond to engine failure incidents that could happen along the flight path. The contingency plans should consist of a set of waypoints and a path information that will guide the UAV to a reachable emergency landing site with a gliding speed in the operational range of the UAV and with respect to the forecast wind conditions. These contingency plans would accompany the primary flight path plans. It is expected that in case of an engine loss, based on where in the flight path the emergency happened, the contingency plan with the contingency point closest to the emergency will be selected, and the UAV by performing the turns in the contingency plan and tracking the waypoints along the contingency plan path, should reach to the designated emergency landing site in the forecast wind conditions.

## 3. Proposed Approach

In the proposed approach, after contingency happens, it is aimed to get the UAV over the airspace of the designated emergency landing site as soon as possible and lose the excessive altitude nearby the designated emergency landing site before conducting the final approach for landing. Because the UAV has no thrust due to engine loss, it is deemed risky to lose excessive altitude away from the landing site which might then result in reachability issues. Moreover, conserving the excess energy in the form of altitude till the last phases of the emergency flight could be advantageous [23]. This can provide extra time to the UAV operator and allow corrections for miscalculations and errors in the contingency plans path and mitigate wind conditions that deviate significantly from the forecast wind.

**Figure 2:**
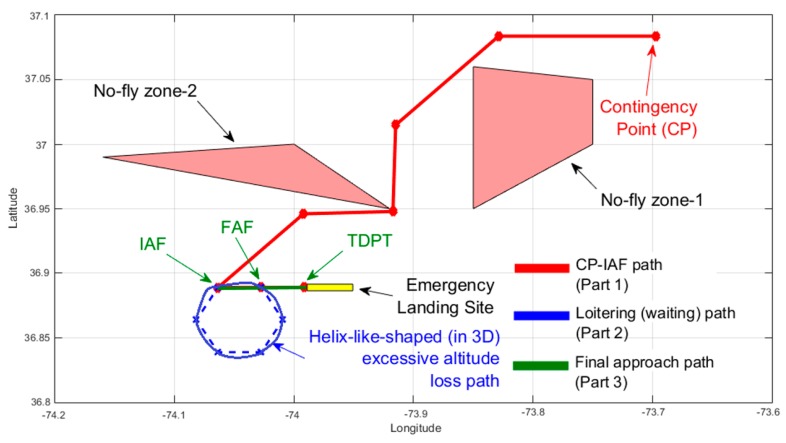
Illustration of the proposed approach on a sample contingency scenario.

An illustration of a preflight contingency plan path is shown in Figure 2. In Figure 2, two no-fly zones can be seen together with the contingency point and the identified emergency landing site. The contingency point itself is a waypoint along the primary flight path and is abbreviated by the notation, CP. The emergency landing site for a contingency plan is selected using the method in [37] with consideration of five safety criterions, the surface type of emergency landing site, and its reachability. Based on the orientation of the selected emergency landing site, three waypoints of the final approach path that form a straight line are identified. They are initial approach fix (IAF), final approach fix (FAF), and touch-down point (TDPT).

A* technique [38] is applied to find a path from CP to IAF. This enables finding a path that avoids the no-fly zones within the theater. The A* path points are sampled such that the turning points are kept as waypoints. The path trajectory between CP and IAF is determined such that wind forecast data is incorporated into the path design with six extremals that are based on trochoidal curves while avoiding the no-fly zones in the theater.

After the path part between CP and IAF is found, the UAV’s altitude at IAF and the altitude-to-be-at for the final approach path are estimated. A total of six heuristic rules are used to plan the rest of the contingency flight path based on these estimations. To meet true reachability, loitering paths to lose the excessive altitude are considered. This is made possible by using a time-constrained path design technique [36] and a look-up table-based methodology. A helix-shaped path is also used to bleed off any significant excessive altitude before the final approach if necessary. The helix-shaped path consists of six waypoints in the shape of a hexagon. From top view, this path looks circular. Two of its six waypoints share the same coordinates of IAF and FAF waypoints as can be seen in Figure 2. Our approach aims a true reachability to the landing place with respect to the gliding speed and forecast wind conditions with the objective to have a tolerable UAV height over TDPT. In summary, the contingency plan path consists of three parts: path from CP to the initial approach fix (IAF); the loitering paths, to lose excessive altitude if necessary; and the final approach path. If there is not enough altitude for the final approach path, an exceptional case is considered in which the UAV directly starts maneuvering towards TDPT from CP as a last resort.

**Figure 3:**
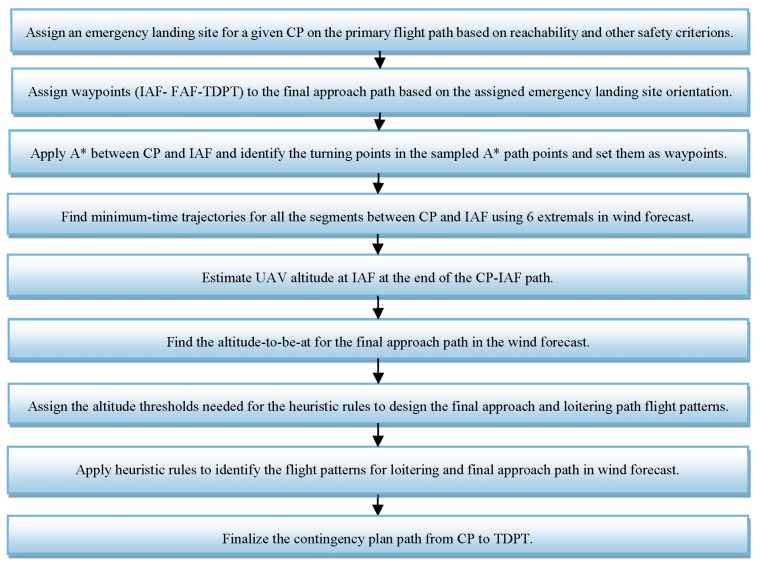
The simplified block diagram of the proposed contingency planning.

Figure 3 shows the simplified block diagram of our preflight contingency planning approach. These blocks will be introduced in detail in the following subsections.

### 3.1. Emergency Landing Site Assignment for a Contingency Point

A primary flight path consists of several pieces of information about the UAV’s flight mission. Amongst them are waypoints along the flight path, the UAV speed and control and guidance values at each leg of the flight path, performance estimates such as fuel consumptions and travel time in each leg of the primary flight path, mission start and end times, takeoff weight value, etc. Joint Mission Planning System (JMPS) [39] is one of the software that can generate primary flight path file. Waypoints of the primary flight path are considered as contingency waypoints. If the distance between any two consecutive contingency waypoints along the flight path is too long, additional contingency waypoints are inserted between those waypoints so that most of the primary flight path is covered in contingency planning. For each contingency waypoint, a contingency plan is generated with the objective to guide the UAV from the contingency point to an identified emergency landing site (crashing/ditching site or local airport runway) in the forecast wind conditions.

In an emergency, the contingency plan is selected based on where the emergency happened on the primary flight path and the closest contingency point to the emergency location. The emergency flight plan of the selected contingency plan is then used for forced landing.

Within the theater of the primary flight path, a list of emergency landing site candidates is identified using our Semi-Automated Emergency Landing Site Selection (SAELSS) method [37]. This method automates the job of finding crashing/ditching sites in a given geographical area of interest. It uses color satellite images, elevation profiles and global human settlement layer to identify a set of landing site candidates and ranks them from the safest to the least safe with respect to five criterions for each surface type and reachability. The dimensions of the landing site are identified based on the UAV size and its landing requirements (minimum runway size, etc.). In addition to emergency landing sites, local airport runways within the theater are also considered as landing site candidates and retrieved from an airport database. In the emergency landing site selection, it is made sure that the airspace over the identified landing sites and a certain area around it do not belong to any no-fly zones. The no-fly zones correspond to airspace that is closed to any type of air traffic due to security and safety reasons. By picking emergency landing sites with no-fly zones in the immediate vicinity, no violation of the no-fly zones is assured during loitering which takes place nearby the emergency landing site. For each contingency waypoint, an emergency landing site is picked among the SAELSS identified landing site candidates.

### 3.2. Waypoint Assignment for the Final Approach Path

The three waypoints of the final approach path (FAP) which are denoted by IAF, FAF and TDPT are identified based on the orientation of the assigned emergency landing site candidate. The three waypoints form a straight line with equal distance from each other. The distance between these waypoints is identified based on the UAV size and performance parameters of the UAV such as the gliding speed. Two of these waypoints (IAF and FAF) are designed to have the same coordinates of the two waypoints of the helix-shaped excessive altitude loss path. This intends to make the IAF waypoint both as the entry point to the helix-shaped excessive altitude loss path and also the exit point from it if there is any significant excessive altitude to bleed off.

### 3.3. Flight Path Design between CP and IAF

The path between CP and IAF forms the first part of the contingency plan path. If there are no-fly zones located between CP and IAF, A* algorithm [38] is applied to find a preliminary path that does not cross through no-fly zones. The number of preliminary A* path points could be numerous. The A* path points are sampled such that the turning points are considered as waypoints while several other A* path points that form straight line-like formations are eliminated. There is no need to apply A* if there is no-fly zone and the final path consists of a single leg that connects CP and IAF waypoints.

**Figure 4:**
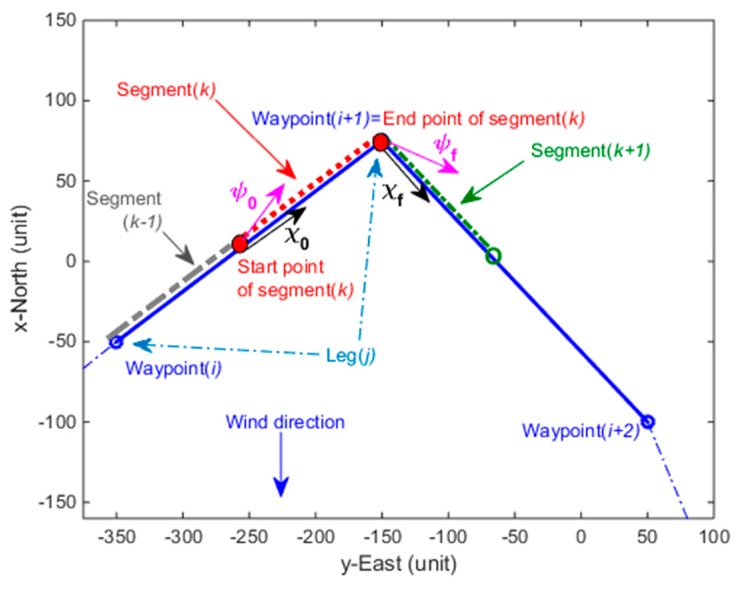
Demonstration of a minimum airtime path for a segment of a leg in the CP-IAF path.

Each connection between two consecutive turning waypoints in the CP-IAF path is considered as a leg. Each leg in the CP and IAF path is partitioned into smaller segments to enable incorporation of varying wind forecast into the overall path design. The objective is to find a minimum-time trajectory based on trochoidal curves for every segment and stitch them together to form the CP-IAF path. Figure 4 shows an illustration of one of the leg segments. The course and heading angles of the UAV at the start and end points of the segment are depicted in Figure 4.

Because the minimum-time trajectory design with trochoidal curves for a segment requires constant wind [34], we assume a steady wind condition within a segment. We consider that the wind variation within a small path segment is negligible in this work. This can be also observed from actual wind forecast data within small regions [40]. The course angle of the UAV at the start of a segment is found in degree clockwise from North using the geographical locations of the start and end of the segments and great circle of the earth. Similarly, the course angle of the UAV at the end of the segment is found using the end of the segment and the start point of the next segment. Using the course angles at the start and end points of the segment, the steady wind amplitude and direction along the segment and the constant gliding airspeed, the headings of the UAV are computed at the start and end points of the segment. The heading angles at the start and end point of the segment are necessary for the minimum-time trajectory design.

**Figure 5:**
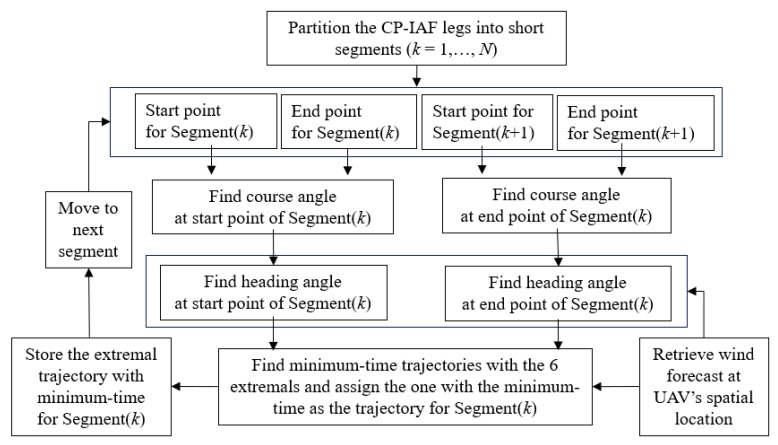
Block diagram showing the process of assigning the minimum-time extremal trajectory to a segment in the CP-IAF path.

Minimum-time trajectory design with trochoidal curves has been studied in [34] with the use of six different extremals. These six extremals are LSL, RSR, RSL, LSR, RLR and LRL. A detailed description of the analytical and numerical solutions for six different extremals can be found in [34]. We compute the minimum-time trajectories for each segment in CP-IAF path using all six different extremals in the forecast wind condition. The path trajectory for a segment is then assigned to the extremal that provides the minimum-time. A block diagram showing the process of assigning the minimum-time extremal trajectory to a segment is shown in Figure 5. A detailed discussion of the CP-IAF path design could be found in our paper [40].

### 3.4. Estimating UAV Altitude at IAF at the End of CP-IAF Path

The UAV altitude at the end of CP-IAF path, *H_IAF_*, is needed to find out the final approach and the excessive altitude loss path patterns. The UAV altitude at the end of each segment within CP-IAF path is found by computing the altitude drops in three phases of the extremal type and subtracting the sum of these three altitude drops from the altitude value at the start point of the segment. Because the travel times in each of the three phases of an extremal type are computed, the altitude drops in each phase can be found using the corresponding sink rate of the UAV in that phase.

Among the six extremal types, some of them contain right and left turns while some has straight level gliding in it. Because sink rates differ when turning and in straight level gliding [24], we considered two different sink rates in the altitude drop computation. Suppose *W* is the aircraft weight at the contingency point, *S* is the wing reference area, *A* is the wing aspect ratio, *C_D_*_0_ is the flags-up parasite drag coefficient, *e* is the airplane efficiency factor, and *V* is the true airspeed of the aircraft. One other parameter to compute UAV’s sink rate is the air density value, *ρ*, at UAV’s altitude. The 1976 COESA air density values at different altitudes are used in this work [41]. The equations for sink rate computation for straight gliding and turning are shown in (1) and (2) [24]. In (1), *V_s_* corresponds to the sink rate for straight level gliding. In (2), *V_sφ_* corresponds to the sink rate when turning. Because a fixed turn rate is considered in the design of the CP-IAF path, the bank angle of the turn, *φ*, can be found using (3):

(1) $$V_{s}=\frac{0.5\rho SC_{D0}}{W}V^{3}+\frac{2V}{\rho S\pi AeV}$$

(2) $$V_{s\phi }=\frac{0.5\rho SC_{D0}}{W}V^{3}+\frac{2V\sec^{2}(\phi )}{\rho S\pi AeV}$$

(3) $$\phi =\tan^{-1}\left(\frac{V\omega }{g}\right)$$

**Figure 6:**
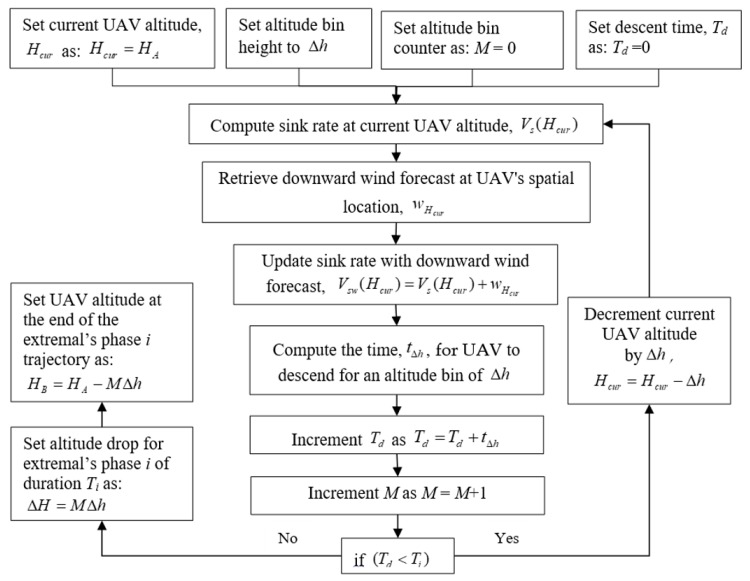
Block diagram of the altitude drop estimation within the *i*-th phase of a minimum-time extremal trajectory when UAV is at altitude *H_A_*.

In (3), ω corresponds to the turn rate in rad/sec and g corresponds to the acceleration of gravity, which is equal to 9.8 m/s^2^. It is worth mentioning that in the sink rate computations, we use the UAV weight at CP. This is the UAV weight obtained by subtracting the estimated weight of the consumed fuel up to CP from the takeoff weight value. The primary flight path obtained with JMPS software provides the takeoff weight value, the type of jet fuel, and the estimated fuel consumption at each leg of the primary flight path. Using this information, we can estimate UAV’s weight at CP. To estimate UAV altitude at IAF, the altitude drops are computed for each of the three phases of the identified extremal type with respect to all the segments in the CP-IAF path. Suppose *T_i_* is the travel time for the *i*-th phase of a minimum-time extremal trajectory assigned to a segment in the CP-IAF path and the UAV’s altitude before starting the *i*-th phase of the minimum-time extremal trajectory is *H_A_*. Suppose the UAV altitude drop that we would like estimate after a travel time of *T_i_* is depicted by Δ*H*. It should be noted that if the *i*-th phase of the extremal type is a turn (right or left), the sink rate equation in (2) for the turn mode is used, and if it is straight level glide, the sink rate equation in (1) for straight level gliding mode is used. For the altitude drop estimation for a single phase of the extremal, first, the sink rate at *H_A_*, is computed which is denoted by *V_s_*(*H_A_*). The downward wind forecast component, $w_{H_{A}}$, at this spatial and altitude location is then added to this sink rate, $V_{sw}\left(H_{A}\right)=V_{s}\left(H_{A}\right)+w_{H_{A}}$. An altitude bin, Δ*h*, is identified and it is assumed that the sink rate, $V_{sw}(H_{A})$, is constant when descending from $H_{A}$ to $H_{A}-\Delta h$. This follows computing the time, $t_{\Delta h}$, for the UAV to descend from $H_{A}$ to $H_{A}-\Delta h$. We store travel time, $t_{\Delta h}$, for this altitude bin. The UAV’s new altitude becomes $H_{A}-\Delta h$. The process of finding the sink rate at the new altitude is repeated while storing the airtime to descend in each new altitude bin. The process is stopped when the sum of stored altitude bin descent times, $T_{d}$, exceed $T_{i}$. Suppose at the *M*th altitude bin, $T_{d}$ exceeds $T_{i}$, the altitude drop, ΔH is then found as ΔH=MΔh and the new altitude value at the end of the *i*-th extremal phase, $H_{B}$, is found as $H_{B}=H_{A}-M\Delta h$. A block diagram showing the altitude drop estimation within the *i*-th phase of a minimum-time extremal trajectory when UAV is at altitude *H_A_*, is shown in Figure 6. The altitude drop estimation process is described in detail in [40] with accompanying pseudocodes.

The altitude drops for each of the three phases of an extremal type trajectory, which correspond to a segment, is found separately and summed to find the overall altitude drop for the segment. The segment altitude drop is then subtracted from the UAV’s altitude value at the start of the segment to initialize the altitude value for the next segment. The minimum time trajectory path estimation and the altitude drop estimation are repeated for all the segments in the CP-IAF path. The sum of the estimated altitude drops for each segment along the CP-IAF is subtracted from the UAV altitude at CP to compute the UAV altitude at the end of CP-IAF path, $H_{IAF}$.

### 3.5. Estimating the Altitude-to-Be-at for the Final Approach Path in the Wind Forecast

The final approach path is designed together with estimating the UAV’s altitude-to-be-at before initiating the final approach path, $H_{FAP}$. The three waypoints of the final approach path lie on a straight line as discussed in the previous section. The first waypoint of the final approach path is IAF. IAF waypoint of the CP-IAF path and IAF waypoint of the final approach path have the same coordinates but different altitudes. The altitude-to-be-at IAF has to be a reasonable one to make the forced landing. We first estimate a rough altitude-to-be-at IAF value for the final approach path, ${\tilde{H}}_{FAP}$, assuming there is no wind. Since the distance between IAF and TDPT, and the constant gliding speed are both known, computing the travel time from IAF to TDPT in no wind assumption is straightforward. Using the estimated travel time between IAF and TDPT, ${\tilde{H}}_{FAP}$ can be approximated. Using ${\tilde{H}}_{FAP}$, we then retrieve the wind components at IAF. Here, we assume that the difference between the wind components at ${\tilde{H}}_{FAP}$ in no wind and actual altitude-to-be-at IAF value are small and negligible. We then re-estimate the travel time from IAF to TDPT and estimate $H_{FAP}$ and the final approach path using the retrieved wind components.

To improve the accuracy of $H_{FAP}$ estimation, one additional process is considered. First, using the final approach path, we check whether the UAV height value at TDPT, $H_{TDPT}$, is tolerable for landing or not. A threshold, $th_{H}$, is set to the maximum tolerable height value. If the absolute value of $H_{TDPT}$ is smaller than $th_{H}$, it indicates that this condition is met in the first iteration and we use the final approach path and $H_{FAP}$ as they are without any modification. If $H_{FAP}$ is not found tolerable for landing, we update $H_{FAP}$ according to the difference between $H_{FAP}$ and $th_{H}$, and redesign the final approach path and re-estimate $H_{FAP}$.

### 3.6. Excessive Altitude Loss Path Design

In this work, we identify the term “excessive altitude” as the difference between the UAV altitude at the end of the CP-IAF path, $H_{IAF}$, and the altitude-to-be-at IAF for the final approach path, $H_{FAP}$, before landing. With the use of heuristic rules, the flight patterns for the excessive altitude loss and the final approach paths are identified. If the excessive altitude is small, the airtime needed to lose the excessive altitude loss is estimated. A time-constrained path design method [36] is used to lose this excessive altitude by setting the estimated airtime as the time constraint. Since there could be cases in which a path that meets the set time-constraint cannot be found, a look-up table-based approach is also considered as an alternative to the time-constrained path method. If the excessive altitude is significant, a helix-shaped path is utilized to bleed the excessive altitude by circling around this path as many times as possible before the final approach path. In the following, we will briefly talk about each of these loitering methods used to lose excessive altitude.

#### 3.6.1. Time-Constrained Path Design

We used [36] to design a time-constrained trajectory path in the presence of wind for an airtime which corresponds to losing a certain amount of excessive altitude. This method assumes a constant airspeed for the UAV and uses steady wind assumption which is in line with our assumptions. The method provides a numerical solution for a time-constrained LRL or RLR type extremal trajectory design in the presence of steady wind conditions for a fixed wing UAV navigating from one point to a nearby point, or back to itself. The trajectory solution in [36] assumes that the UAV has the same turn rates for the second and third turns of the extremal while the first turn can have a different turn rate between the minimum and maximum turn rate. The second order Newton-Raphson method is used in this method for solving time-constrained extremals.

#### 3.6.2. Look-up Table-Based Time-Constrained Path Design

In case time-constrained path design method fails to provide a solution, a look-up table (LUT) based approach is used. The generated look-up table consists of trajectories and their air times from six different extremal types (LSL, RSR, RSL, LSR, LRL, and RLR) of which the heading at the start and at the end of turn are same. In generating look-up-table, the considered wind direction has a range from 0° to 359° with a degree step size of 1°. The wind amplitude has a range from 0 to 50 m/s with a step size of 1 m/s. The turn rate is considered from 0.01 rad/s to 0.06 rad/s with a step size of 0.001. These trajectories and their airtimes are computed with consideration of the performance parameters of the UAV. Suppose a loitering path is needed with a specific airtime for a wind direction and amplitude with the same heading values at the start and end points. From the look-up table, the extremal trajectory that has a travel time closest to the requested airtime in the similar wind condition is picked as the loitering path trajectory.

#### 3.6.3. Helix-Shaped Excessive Altitude Path Design

The path to lose significant excessive altitudes is a helix-shaped path nearby the emergency landing site which consists of six waypoints in the shape of a hexagon as is shown in Figure 2. Two waypoints of the helix-shaped path have the same coordinates of IAF and FAF, which are the two waypoints in the final approach path. If the excessive altitude is significant, this helix-shaped path is used to bleed the excessive altitude as much as possible by taking several cycles around this path. If there is still some excessive altitude which is not enough to lose with one other circling of the helix-shaped path, the remaining excessive altitude is then lost using either the time-constrained path method or the look-up table-based method like before. The distance between two consecutive waypoints of the helix-shaped path is same and is equal to the distance between IAF and FAF waypoints of the final approach path.

### 3.7. Assigning Altitude Thresholds for Heuristic Rules

A total of six heuristic rules are used to identify the final approach path and excessive altitude loss path patterns based on the estimated altitude at IAF after the CP-IAF path, $H_{IAF}$, and the altitude-to-be-at IAF right before the final approach path, $H_{FAP}$. To decide which heuristic rule to apply, five altitude thresholds which have the unit of feet, $th_{0}$, $th_{1}$, $th_{2}$, $th_{3}$ and $th_{4}$ are assigned by considering several factors such as the UAV size, gliding speed, the distance between IAF and FAF, the total length of the helix-shaped like excessive altitude loss. The altitude of UAV at IAF, $H_{IAF}$, are compared with these five altitude thresholds to finalize the flight pattern of the final approach path and the loitering paths to lose excessive altitude if any. Among these thresholds, $th_{0}$ corresponds to the case where the UAV does not have enough altitude to complete the final approach path after arriving IAF from CP. Suppose $H_{ELS}$ is the elevation of the emergency landing site. We set $th_{0}$ as follows:

(4) $$th_{0}=\left(H_{FAP}-H_{ELS}\right)/3+H_{ELS}$$

$th_{1}$ and $th_{2}$ correspond to the case in which $H_{IAF}$ is almost equal to $H_{FAP}$. For this case the UAV directly follows the final approach path after the CP-IAF path without any need to lose excessive altitude. We set $th_{1}$ and $th_{2}$ as follows: $th_{1}=H_{FAP}-150$ and $th_{2}=H_{FAP}+150$. $th_{3}$ corresponds to the case where the UAV does not have enough excessive altitude to complete one cycle of the helix-shaped excessive altitude loss path and the excessive altitude is relatively small. We set $th_{3}$ as follows:

(5) $$th_{3}=2.5\left(H_{FAP}-H_{ELS}\right)+H_{ELS}$$

**Figure 7:**
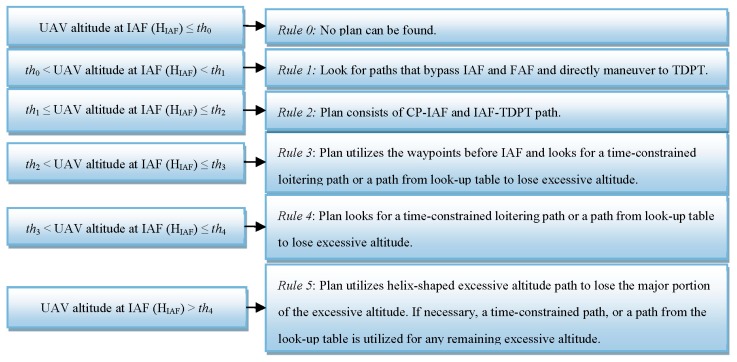
Block diagram for the heuristic rules.

Suppose $H_{E}$ is the estimated altitude lost value that corresponds to one cycle of rotation of the UAV around the helix-shaped excessive altitude loss path right before reaching $H_{FAP}$ in no wind condition with some buffer height added to compensate for wind and air density variation effects. We set $th_{4}$ as follows: $th_{4}=H_{E}+H_{FAP}$. The block diagram in Figure 7 shows how these altitude thresholds are used with the six heuristic rules which will be introduced in the next section.

### 3.8. Heuristic Rules to Finalize Excessive Altitude Loss and Final Approach Patterns

Once $H_{IAF}$ and $H_{FAP}$ are estimated, the next step is to identify the flight patterns for the final approach path and loitering paths depending on the excessive altitude value. We identify six heuristic rules which are introduced in the following and demonstrated with a block diagram in Figure 7.

*Rule-0* ($H_{IAF}\leq th_{0}$): This corresponds to the case where there is not enough excessive altitude at the end of CP-IAF path to complete the final approach path. A contingency plan can’t be found.

*Rule-1* ($th_{0}<H_{IAF}<th_{1}$): For cases where there is not enough excessive altitude to fully complete final approach path, true reachability could be still possible if final approach path is not followed but rather the UAV turns directly towards TDPT (bypassing the IAF and FAF waypoints) while it is still somewhere on the CP-IAF path. In Case-1, several path candidates are considered. This involves searching for a waypoint along CP-IAF path to make a forced landing from this waypoint to TDPT bypassing IAF and FAF. If there is not enough number of waypoints on the CP-IAF path (which indicates a low altitude emergency), it is then searched for a path from CP to TDPT via a time-constrained path or with one of the six extremal types in the trajectory look-up table with a range of different turn rates. Among the candidates, final approach path is assigned to the one providing a final height value that is closest to 0 and has a positive sign. In Rule-1, it is also assumed that the UAV can cross through no-fly zones to make a forced landing as a last resort.

*Rule-2* ($th_{1}\leq H_{IAF}\leq th_{2}$): If $H_{IAF}$ takes a value between $th_{1}$ and $th_{2}$, UAV follows the final approach path after CP-IAF path without any attempts to lose excessive altitude since the excessive altitude to lose is negligible. That is, $H_{IAF}$, is almost equal to $H_{FAP}$.

*Rule-3* ($th_{2}<H_{IAF}\leq th_{3}$): This rule is for the case where there is some marginal excessive altitude at IAF after the CP-IAF path is completed, but it is not significant enough for a helix-shaped excessive altitude loss path. This rule considers the waypoint before IAF (which is denoted by BIAF) and aims path extension possibilities from BIAF to IAF or BIAF to FAF using the time-constrained path design method to fulfill reachability. If no time-constrained paths can be found, it then looks for an extremal path trajectory from BIAF to IAF with one of the six extremals in the extremal trajectory look-up table. The path extension is included to form the whole contingency plan path from CP to TDPT.

*Rule-4* ($th_{3}<H_{IAF}\leq th_{4}$): This heuristic is for the case where there is considerable excessive altitude at IAF after the CP-IAF path is complete. The excessive altitude is not enough to use the helix-shaped excessive altitude loss path, but it is enough to consider a loitering path that will make the UAV return to the same starting point (IAF) with the same heading angle. Like Rule-3, this heuristic first looks for possible loitering paths from IAF to itself using time-constrained path design method to meet reachability. If no time-constrained paths can be found, it then picks an extremal path from the trajectory look-up table with the closest airtime to lose the excessive altitude. The loitering paths are connected to form the whole contingency plan path from CP to TDPT.

*Rule-5* ($th_{4}<H_{IAF}$): If $H_{IAF}$ is bigger than $th_{4}$, the helix-shaped excessive altitude loss path is cycled as many times as possible. Since the helix-shaped excessive altitude loss path consists of multiple segments, it is formed in the same way the CP-IAF path is formed with the 6 extremals based on trochoidal curves. After the complete of each cycle of the helix-shaped altitude loss path and arriving back at IAF, $H_{IAF}$ is updated accordingly with the altitude lost value for the computation of the next cycle. After all possible cycles are completed, if the remaining excessive altitude is bigger than $th_{3}$, like Case-4, it is first looked for possible path extensions from IAF to itself using time-constrained path design method with the estimated airtime to lose the remaining excessive altitude. If no time-constrained path is found, it then looks for a path from the trajectory look-up table of which its travel time is closest to the required airtime. If, on the other hand, the remaining excessive altitude (after all cycles are completed) is less than $th_{3}$, the last cycle of the hexagonal-shaped excessive altitude loss path is assumed as if it is not included and its corresponding altitude lost value is added to $H_{IAF}$. This makes $H_{IAF}$ > $th_{4}$. The excessive altitude which corresponds to $H_{IAF}-H_{FAP}$ is then partitioned into two equal altitudes. For each partition, either a time-constraint path is used, or an extremal type from the look-up table (LUT). The overall path then consists of the following parts: (a) CP-IAF, (b) IAF-IAF (helix-shaped excessive altitude loss path, could be multiple cycles), (c) IAF-IAF loitering path (time-constrained path or an extremal trajectory from the look-up table), and (d) Final approach path (IAF-FAF-TDPT).

## 4. Results

In the simulations, we used our own gliding flight simulation model which can retrieve wind forecast data at each new spatial location of UAV and we considered a UAV with the aerodynamic specifications shown in Table 1.

**Figure 8:**
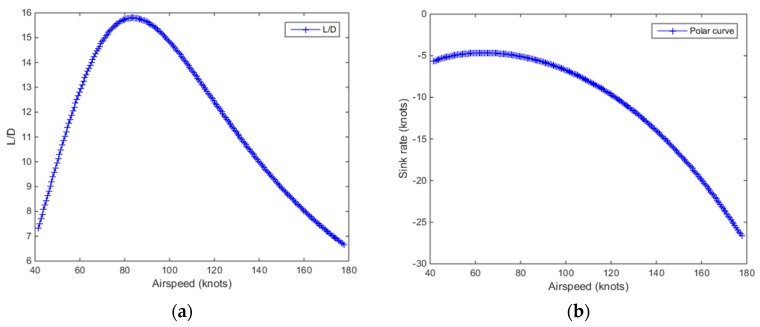
(**a**) L/D curve and (**b**) polar curves for the hypothetical UAV used in the simulations.

The hypothetical UAV specifications were obtained from the internet and have some resemblances to those of a MQ-4C Triton surveillance aircraft. However, it should be noted that our framework can be adapted to other manned aircraft such as the Airbus 320. An example will be mentioned later in this paper. The resultant polar curve and Lift/Drag (L/D) ratio curve of the hypothetical UAV are shown in Figure 8. It is assumed that the UAV has a constant gliding true airspeed of 140 knots, *V* = 140 knots. From the UAV’s polar curve at this gliding speed, the UAV has a sink rate of 14 knots, $V_{s}=14$ knots, at 4000 feet. It is worth mentioning that the sink rate changes according to the altitude of the aircraft since the air density varies at different altitudes and sink rate computation is dependent on the air density. Our method incorporates varying air density at different altitudes and computes the sink rates accordingly.

The distance between the waypoints in the final approach path (IAF-FAF and FAF-TDPT) is set to 2 miles. This automatically sets the distance between two consecutive waypoints of the helix-shaped excessive altitude loss path to 2 miles since IAF and FAF are two of the six waypoints in the helix-shaped excessive altitude loss path. The CP-IAF path is partitioned into shorter segments with 2 miles each. The altitude bin, Δh, in altitude drop estimation is set to 5 ft.

For wind forecast data, we used the hourly-updated wind forecast data from National Oceanic & Atmospheric Administration’s (NOAA) National Centers for Environmental Prediction (NCEP) with respect to the theater and the mission start and end times [42]. The wind forecast data is possible by Rapid Refresh (RAP) which is the continental-scale hourly-updated assimilation/ modeling system operational at NCEP [43]. RAP forecasts are generated every hour with forecast lengths going out 18 h. A program called “wgrib2” is used to extract the compressed GRIB2 files and retrieve the data contents for the theatre of interest [44]. With “wgrib2” program, we can retrieve the wind velocities and their angular directions for the theater zone of interest at 37 different vertical pressure levels (100 mb to 1000 mb) where the pressure in millibar can be converted to pressure altitude. The wind forecast data at two of these pressure altitude levels that are closest to the aircraft altitude are interpolated to estimate the wind forecast data at the UAV altitude. The wind forecast data at two different altitudes used in the simulations in this work corresponds to the date of 19 June 2017 at 11:00 a.m. The wind forecast data is not time-varying within the same hour; however, it is spatially varying in three dimensions both horizontally and vertically. Plots of the wind profiles used in this work can be found in [40].

In the following results which demonstrate five cases in the forecast wind with respect to the heuristic rules, we have set the altitude thresholds as follows: $th_{0}$ = 715 ft, $th_{1}$ = 1995 ft, $th_{2}$ = 2295 ft, $th_{3}$ = 5362 ft and $th_{4}$ = 11,690 ft. It is worth mentioning that the assigned altitude thresholds for the six rules and other parameters in this work are for demonstration purposes only for the UAV of interest and they should be revisited by the mission planners depending on the specifications of the aircraft of their interest.

**Figure 9:**
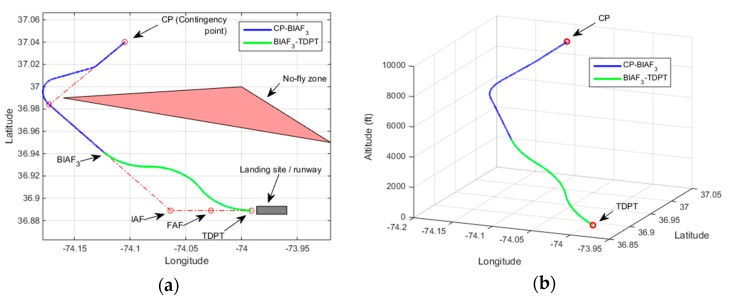
Demonstration of Rule-1 heuristic with no crossing through the no-fly zone scenario, Altitude at CP = 9000 ft, Estimated altitude at IAF = 1575 ft, Height over TDPT = 30 ft (**a**) 2D plot; (**b**) 3D plot.

**Figure 10:**
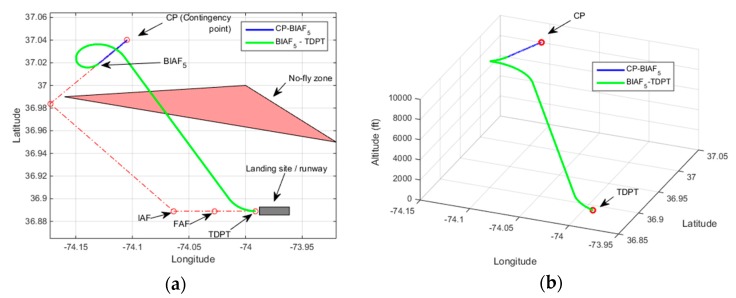
Demonstration of Rule-1 heuristic with crossing through the no-fly zone scenario, Altitude at CP = 8510 ft, Estimated altitude at IAF = 885 ft, Height over TDPT = 15 ft (**a**) 2D plot; (**b**) 3D plot.

Figure 9 and Figure 10 correspond to demonstrations of Rule-1 for two different scenarios. In Figure 9 and Figure 10, the resultant contingency plan paths are shown both in 2D and 3D plots. The no-fly zone is shown only in the 2D plot and the contingency plan path alone is shown in the 3D plot. In the first scenario, the UAV altitude at CP is 9000 ft. The elevation of the emergency landing site is 0 ft. The UAV altitude at IAF (after the CP-IAF path) in the wind forecast is estimated to be 1575 ft which triggers Rule-1. The resultant contingency flight path as is shown in Figure 9 does not cross through the no-fly zone and the UAV maneuvers directly to TDPT from $BIAF_{3}$, where $BIAF_{3}$ corresponds to the third waypoint before IAF in the CP-IAF path. The UAV height over TDPT is estimated to be 30 ft which is considered within the tolerable height range for landing. In the second scenario shown in Figure 10, the UAV altitude at CP is 8510 ft. The estimated UAV altitude at IAF is 885 ft. Because there is not enough airtime to track the final approach path after the completion of the CP-IAF path, the UAV maneuvers to TDPT from $BIAF_{5}$ (fifth waypoint before IAF in the CP-IAF path) bypassing the final approach path and the IAF and FAF waypoints. The UAV height over TDPT is estimated to be 15 ft. In the second scenario, even though a contingency flight path is found, it is observed that it crosses the no-fly zone as a last resort to fulfill reachability.

**Figure 11:**
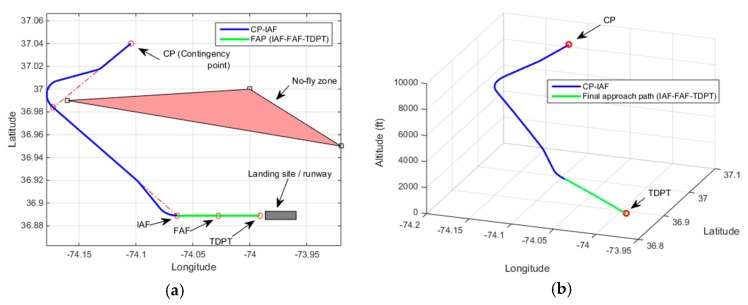
Demonstration of Rule-2, Altitude at CP = 9400 ft, Estimated altitude at IAF = 2120 ft, Height over TDPT = 15 ft (**a**) 2D plot; (**b**) 3D plot.

**Figure 12:**
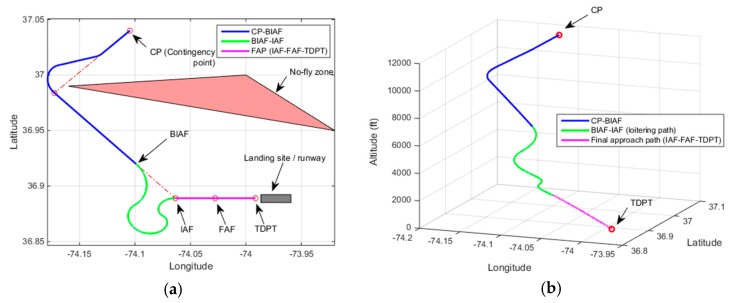
Demonstration of Rule-3, Altitude at CP: 12,000 ft, Estimated altitude at IAF = 5355 ft, Height over TDPT = 10 ft (**a**) 2D plot; (**b**) 3D plot.

Figure 11 demonstrates a contingency plan path found with Rule-2 heuristic. For this demonstration, the UAV altitude at CP is set to 9400 ft. The estimated altitude at IAF is 2120 ft and the estimated final height of the UAV over TDPT is 15 ft. No loitering path design is needed. The overall contingency plan path consists of: (a) CP-IAF path, (b) Final approach path (FAP). Figure 12 demonstrates the contingency plan path with Rule-3. The UAV altitude at CP is set to 12,000 ft. The estimated altitude at IAF is 5355 ft which triggers Rule-3 heuristic. The final height of the UAV over TDPT is estimated to be 10 ft.

**Figure 13:**
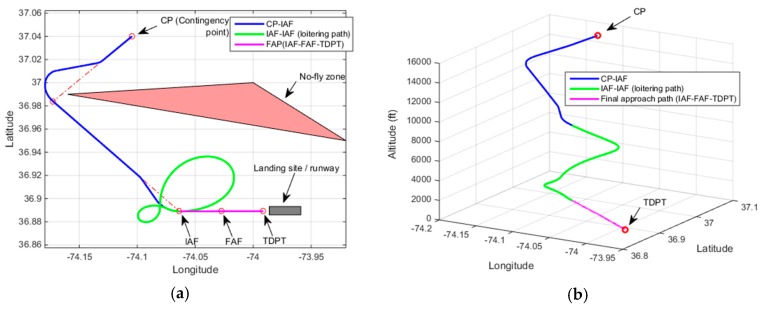
Demonstration of Rule-4, Altitude at CP: 16,000 ft, Estimated altitude at IAF = 9825 ft, Height over TDPT = 45 ft (**a**) 2D plot; (**b**) 3D plot.

Figure 13 demonstrates a contingency flight path as a result of Rule-4 activation. In this demonstration, the UAV altitude at CP is 16,000 ft. The estimated altitude at IAF is found to be 9825 ft. The final height of the UAV is estimated to be 45 ft. A loitering path to lose a minor excessive altitude can be observed as a part of the resultant contingency plan. This loitering path is found with the time-constrained based trajectory method.

**Figure 14:**
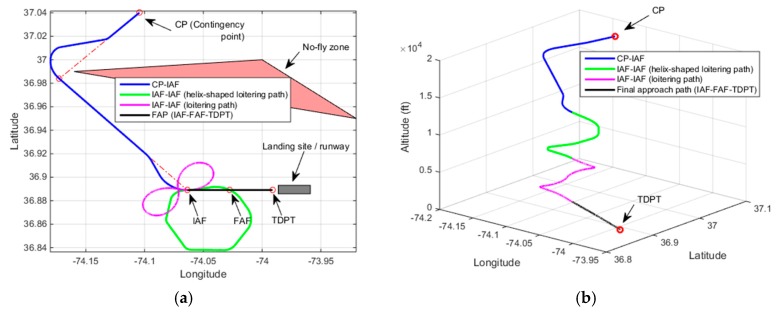
Demonstration of Rule-5, Altitude at CP: 20,000 ft, Estimated altitude at IAF = 14,160 ft, Height over TDPT = 20 ft (**a**) 2D plot; (**b**) 3D plot.

Figure 14 demonstrates a contingency plan path generated with Case-5 heuristic. In the demonstration, the UAV altitude at CP is 20,000 ft. The estimated altitude at IAF is 14,160 ft. The final height of the UAV over TDPT is estimated to be 20 ft. A helix-shaped excessive altitude loss path is utilized in the contingency plan path since the excessive altitude to lose after arriving at IAF from CP is significant. The height of the UAV over TDPT is estimated to be a tolerable height of 20 ft for landing.

**Figure 15:**
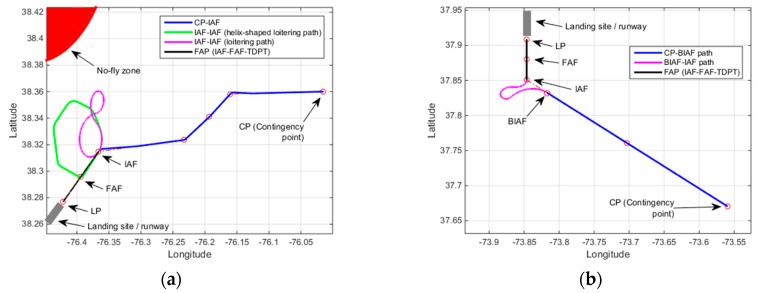
Additional demonstrations (**a**) Altitude at CP = 24,964 ft, Estimated altitude at IAF = 14,129 ft, Altitude over LP = 49 feet (Rule-5 is activated), elevation = 39 feet, height = 10 feet; (**b**) Altitude at CP = 20,219 ft, Estimated altitude at IAF = 5144 ft, Altitude over TDPT = 19 feet (Rule-3 is activated), elevation = 0 feet, height = 19 feet.

Two additional contingency plan demonstrations of the same UAV with two different emergency landing sites are discussed in the following. In the first demonstration as is shown in Figure 15a, the altitude of the UAV at CP is set to 24,964 ft. In this demonstration, Rule-5 is activated since the estimated altitude of the UAV at IAF (after CP-IAF path) is 14,129 ft. The designed contingency plan path consists of a helix-shaped excessive altitude loss path and another RLR extremal type loitering path before having the final approach to the emergency landing site. The elevation of the emergency landing site is 39 feet and the estimated height of the UAV over TDPT with the forecast wind for the area is estimated to be 10 feet. In the second demonstration in Figure 15b, the altitude of the UAV at CP is set to 20,219 ft. Rule-3 is triggered in this demonstration since the altitude at IAF is estimated to be 5144 ft. The runway elevation is 0 ft and the estimated height of the UAV over TDPT is found to be 19 ft.

Table 2 and Table 3 contain detailed information about the resultant contingency plan path of the first demonstration in Figure 15a. In Table 2, the extremal types, their corresponding airtimes and turn rates in all three phases along each segment of the formed overall contingency plan flight path are shown. The start and end coordinates in each segment can be seen in Table 2 as well. Table 3 contains information about the wind forecast along each segment of the contingency plan path. These wind values belong to the start point of each segment in the path since a steady wind condition is assumed along a segment. Table 3 contains the heading values at the start and end of the segments. It could be noticed from these heading values that in the transition from one segment to the next one, there could be small discrepancies. This is because of the steady wind assumption within a segment and facing a slightly varying wind condition in the next segment. It is assumed that the effects of these small discrepancies are negligible, and the UAV can compensate for these undesired effects using its flight control surfaces.

**Figure 16:**
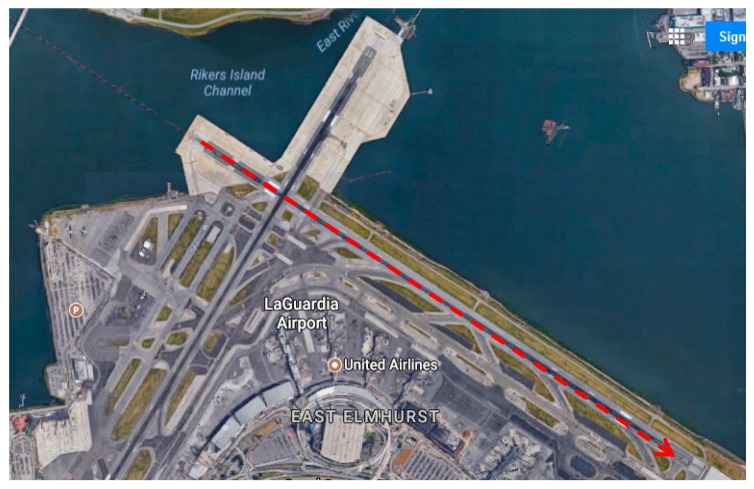
Targeted runway for emergency landing for the Hudson River incident (image is from [48]).

We also applied our method to the flight data collected from an actual incident. US Airways Flight 1549, which was an Airbus A320, was on route from New York City’s LaGuardia Airport to Seattle, Washington and ditched into Hudson River on 15 January 2009. In the climb out phase right after its takeoff, it struck a flock of Canada geese and lost its engine power. The pilots Mr. Sullenberger and Mr. Skiles glided the plane to a ditching in the Hudson River. Everyone was rescued by nearby boats. We used this incident to test our contingency plan method. We found the flight profile which contained the coordinates and altitude of the Flight 1549’s emergency landing path from [45]. The coordinates and altitudes information from takeoff to ditching into Hudson were stored in a KML file [45]. At the time of the bird strike, Flight 1549’s airspeed was about ~200 knots [46]. The highest altitude right before the plane started sinking was 3034 feet (925 m). At this altitude the plane was located at coordinates: Latitude: 40.861666°, Longitude: −73.879722°. Time was 3:27:29 p.m. [45]. The recorded plane coordinates in the KML file right before reaching highest altitude was: Latitude: 40.858055°, Longitude: −73.879027° and the altitude at this location was 923 feet [45]. The wind amplitude was around 13.4 knots. The wind direction was 320° [47]. Assuming the coordinates at the highest altitude corresponds to the coordinates of CP, we found the heading angle at CP using the waypoint coordinates at the highest altitude, the waypoint right before that, and the wind information. The coordinates of the targeted emergency runway that is considered to land back on LaGuardia airport are found from Google Maps [48]. The start and end of the runway had the following coordinates: Start of the runway: Latitude: 40.782344°, Longitude: −73.878641°, End of the runway: Latitude: 40.776786°, Longitude: −73.866900°. The map of the targeted emergency runway is shown in Figure 16. Elevation of LaGuardia Airport is set to ~20 feet [49]. Using wind information and the targeted runway coordinates; we find the heading angle of the plane for landing. We used the aerodynamic parameters of Airbus A320 that was used in [24]. These parameters can be seen in Table 4.

**Figure 17:**
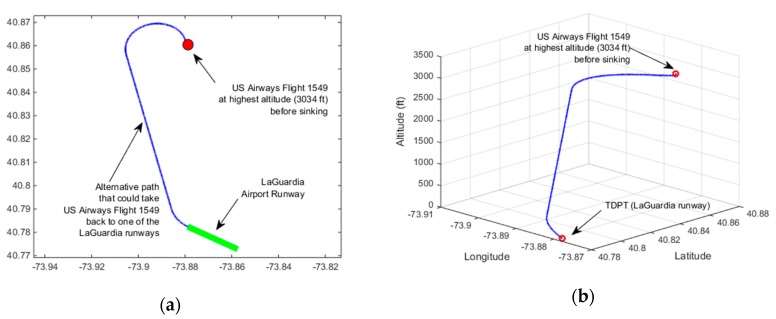
Emergency landing path for Hudson River incident found with our approach (**a**) 2D plot (**b**) 3D plot.

Rule-1 heuristic was invoked with our method when finding the contingency path for emergency landing. This corresponds to low altitude emergency and it bypasses IAF and FAF and attempts to reach TDPT directly from CP. In the simulation, it is assumed that the gliding speed of the aircraft is 182 knots and the maximum turn rate is 0.08 rad/s. The contingency plan path found is shown in Figure 17. It is observed that a flight path that can reach to one of LaGuardia’s runways could be possible in the observed wind condition. The final height of the plane when it reaches to the runway is estimated to be +4 feet. If an automated contingency planning system had been available whether onboard or in a ground control station in communication with the aircraft, the pilots would have been equipped with more information that will help in their decision to whether take the plane back to airport or consider crash landing or ditching.

## 5. Conclusions

In this work, we introduce an approach for preflight contingency planning that automatically generates flight paths for UAVs with full loss of thrust to land them at an emergency landing site without violating no-fly zones in the area and by integrating wind into the path planning process. Loitering paths are used when there is excessive altitude to lose via a time-constrained path design or picking an extremal trajectory path from a look-up table of which its travel time is closest to the required airtime to bleed off the excessive altitude. The proposed approach aims true reachability by providing a safe final altitude value for the UAV to make its final approach before emergency landing in the forecast wind conditions. Instead of Dubins curves, trochoidal trajectories in the form of six extremal types are utilized in the contingency plan path. The proposed preflight contingency planning approach can be an important tool to support the UAV operators if an engine loss incident happens. Moreover, in a situation which the communication link between the UAV and operator is lost, the UAV is all by itself and needs to autonomously land itself to an emergency landing site. With a preflight contingency plan already uploaded to the UAV, with some minor revisions using the inputs from UAV’s onboard sensors, the UAV can utilize this preflight contingency plan to make a forced landing autonomously.

There are some assumptions made in this work. One of them is the steady wind assumption in a short segment of the path. Even though the wind amplitude and direction could be close to each other in value in consecutive segments of the path, there is minor discontinuity when moving from one segment to next one. These minor differences could add up and affect the overall reachability. One of the areas to improve would be about how the slightly varying wind condition within a segment could be addressed. It is also assumed that the transition to turning flight occurs instantaneously instead of accounting for the time that it would take to acquire the target bank angle and turn rate. This is another area in which we want to incorporate the transition times into the path design. Our approach accounts for the fixed no-fly zones. We would like to consider the topography of the area in the future contingency plan path design. As an example, there could be a mountainous area in the area which is not labeled as a no-fly zone and could pose some danger to the UAV if is located between the emergency landing site and the contingency point. By including topography in the path design, these challenges can be overcome. A further objective is to also incorporate dynamically changing threats in the contingency planning if there is some sort of prediction of their time-space trajectories. Examples to dynamically changing threats could be a fast-moving storm, hurricane, microburst or flock of flying birds. Finally, we consider transforming our approach to an in-flight one which utilizes real-time onboard sensor feedback for contingency planning and test its feasibility with realistic flight simulators and actual flight experiments in varying wind conditions.

## Figures and Tables

**Table 1:** Aerodynamic specifications of the hypothetical UAV used in the simulations.

Specs	Notation	Value	Unit
Aircraft Weight (at emergency point)	*W*	32,250	lbf
Wing reference area	*S*	685.3924	*ft* ^2^
Wing aspect ratio	*A*	25	unitless
Parasite drag coefficient (flags up)	*C_D_*_0_	0.0708	unitless
Airplane efficiency factor	*e*	0.9	unitless

**Table 2:** Turn times of the extremal types along the contingency plan path of the first demonstration.

Seg. No	Latitude (Start)	Longitude (Start)	Latitude (End)	Longitude (End)	Turn Type	T1 (s)	T2 (s)	T3 (s)	Total (s)	*ω*_1_	*ω*_2_	*ω*_3_
1	38.3601	−76.0149	38.3596	−76.0518	LSR	0.002	68.341	0.002	68.345	0.06	0.06	0.06
2	38.3596	−76.0518	38.3592	−76.0887	LSR	0.002	67.428	0.002	67.433	0.06	0.06	0.06
3	38.3592	−76.0887	38.3587	−76.1256	LSR	0.002	67.537	0.002	67.542	0.06	0.06	0.06
4	38.3587	−76.1256	38.3583	−76.1604	RSL	0.543	55.240	8.201	63.985	0.06	0.06	0.06
5	38.3583	−76.1604	38.3411	−76.1930	LSR	0.018	55.716	1.282	57.015	0.06	0.06	0.06
6	38.3411	−76.1930	38.3236	−76.2331	LSR	0.235	63.179	5.829	69.243	0.06	0.06	0.06
7	38.3236	−76.2331	38.3212	−76.2698	LSR	0.002	65.435	0.002	65.440	0.06	0.06	0.06
8	38.3212	−76.2698	38.3188	−76.3066	LSR	0.002	64.442	0.002	64.447	0.06	0.06	0.06
9	38.3188	−76.3066	38.3148	−76.3659	RSL	0.468	90.833	9.581	100.882	0.06	0.06	0.06
10	38.3148	−76.3659	38.2959	−76.3938	LSR	1.662	37.752	8.718	48.132	0.06	0.06	0.06
11	38.2959	−76.3938	38.3053	−76.4287	RSR	6.637	49.711	6.165	62.513	0.06	0.06	0.06
12	38.3053	−76.4287	38.3337	−76.4357	RSR	6.177	45.553	7.427	59.157	0.06	0.06	0.06
13	38.3337	−76.4357	38.3527	−76.4079	RSR	7.640	27.568	10.351	45.559	0.06	0.06	0.06
14	38.3527	−76.4079	38.3433	−76.3730	RSR	9.648	16.257	11.613	37.518	0.06	0.06	0.06
15	38.3433	−76.373	38.3148	−76.3659	RSR	3.143	13.405	26.216	42.765	0.06	0.06	0.06
16	38.3148	−76.3659	38.3148	−76.3659	RLR	106.966	89.362	34.180	230.507	0.031	0.06	0.06
17	38.3148	−76.3659	38.2959	−76.3938	LSL	0.003	50.772	0.001	50.777	0.06	0.06	0.06
18	38.2959	−76.3938	38.2769	−76.4216	LSR	0.003	46.701	0.003	46.708	0.06	0.06	0.06

**Table 3:** Wind forecast along the contingency plan path of the first demonstration.

Seg. No	Altitude Start (ft)	Altitude End (ft)	Heading Start (Rad)	Heading End (Rad)	Wind Amplitude (Knots)	Wind Direction (Degree)	Downward Wind Amplitude (Knots)
1	24,964	23,874	5.0246	5.0246	61.6270	316.3931	−0.1513
2	23,874	22,784	5.0202	5.0202	60.5172	316.7610	−0.1422
3	22,784	21,669	5.0208	5.0206	60.6834	316.7443	−0.1230
4	21,669	20,584	5.0164	4.5569	60.3718	316.2959	−0.1111
5	20,584	19,604	4.5594	4.6353	60.7660	315.9590	−0.0863
6	19,604	18,389	4.6368	4.9724	60.8853	316.2661	−0.0676
7	18,389	17,219	4.9683	4.9683	59.7897	316.7280	−0.0300
8	17,219	16,039	4.9658	4.9658	58.7207	317.5811	−0.0137
9	16,039	14,129	4.9423	4.3955	54.6326	317.8300	−0.0016
10	14,129	13,184	4.3955	4.8189	47.6111	317.1416	0.003
11	13,184	11,934	4.7863	5.5545	43.2261	315.4220	0.0001
12	11,934	10,719	5.5479	0.0809	39.5786	313.7011	2.79 × $10^{-5}$
13	10,719	9754	0.0979	1.1774	36.7210	312.5690	−0.00055
14	9754	8944	1.1912	2.4669	34.5778	312.0465	−0.00663
15	8944	7999	2.4699	4.2315	33.02892	310.9417	−0.00163
16	7999	2549	4.2249	4.2249	32.36574	308.3569	−0.00148
17	2549	1264	3.9731	3.9729	17.92907	218.1913	0.04523
18	1264	49	3.8892	3.8892	16.2911	161.0275	0.0150

**Table 4:** Aerodynamic parameters of the Airbus 320 used in the simulations.

Specs	Symbol	Value	Unit
Aircraft sink (at emergency point)	*W*	150,871	lbf
Wing reference area	*S*	1318.579	*ft* ^2^
Wing aspect ratio	*A*	9.5	unitless
Parasite drag coefficient (flags up)	*C_D_*_0_	0.022	unitless
Airplane efficiency factor	*e*	0.7697	unitless

## Abbreviations

The following abbreviations are used in this manuscript:

BIAF	Waypoint before IAF in the CP-IAF contingency plan path
CP	Contingency waypoint
CP-IAF	Contingency plan path section from CP to IAF
FAP	Final Approach Path
FAF	Final Approach Fix waypoint
IAF	Initial Approach Fix waypoint
JMPS	Joint Mission Planning System
LRL	Left-Right-Left extremal
LSL	Left-Straight-Left extremal
LSR	Left-Straight-Right extremal
PFP	Primary Flight Path
RAP	Rapid Refresh
RLR	Right-Left-Right extremal
RSL	Right-Straight-Left extremal
RSR	Right-Straight-Right extremal
SAELSS	Semi-Automated Emergency Landing Site Selection
TDPT	Touchdown waypoint
UAV	Unmanned aerial vehicle
**Nomenclature**	
*A*	Wing aspect ratio,
*C_D_*_0_	Flags-up parasite drag coefficient
*e*	Airplane efficiency factor.
*g* (m/s^2^)	Acceleration of gravity, 9.8 m/s^2^
*H_A_* (ft)	UAV altitude before the *i*-th phase of a minimum-time extremal trajectory
*H_B_* (ft)	UAV altitude after the *i*-th phase of a minimum-time extremal trajectory
*H_E_* (ft)	Estimated altitude lost value during one cycle around the helix-shaped excessive altitude loss path before descending to in no wind condition
*H_IAF_* (ft)	UAV altitude at the end of CP-IAF path section of the contingency plan
*H_ELS_* (ft)	Elevation of the emergency landing site
*H_FAP_* (ft)	Altitude-to-be-at for the UAV before initiating the final approach path
${\tilde{H}}_{FAP}$ (ft)	Approximate altitude-to-be-at for the UAV before the final approach path with no wind assumption
*H_TDPT_* (ft)	UAV altitude estimate over TDPT in the contingency plan
*M*	Altitude bin counter value used in estimating the altitude drop
*S* (ft^2^)	Wing reference area,
*t_A_* (s)	UAV takeoff time from point *A*
*t_B_* (s)	UAV estimated landing time at point *B*
*t*_Δ_*_h_* (s)	UAV descent time within a single altitude bin height
*th_H_* (ft)	Maximum tolerable height value over TDPT for a contingency plan
*th*_0_ (ft)	Threshold used in the heuristic rule that corresponds to the case where the UAV does not have enough altitude to complete the final approach path after arriving at IAF from CP.
*th*_1_, *th*_2_ (ft)	Thresholds used in the heuristic rule that corresponds to the case in which *H_IAF_* is almost equal to *H_FAP_* and there is no need for excessive altitude loss path
*th*_3_ (ft)	Threshold used in the heuristic rule that corresponds to the case where the UAV has relatively small excessive altitude, not enough to complete one cycle of the helix-shaped excessive altitude loss path.
*th*_4_ (ft)	Threshold used in the heuristic rule that corresponds to using the helix-shaped excessive altitude loss path due to significant excessive altitude.
*T_i_* (s)	Travel time for the *i*-th phase of a minimum-time extremal trajectory assigned to a segment in the CP-IAF path.
*T_d_* (s)	Accumulated altitude bin descent times used in altitude drop estimation
*V*	UAV true airspeed
*V_s_* (knots)	UAV sink rate for straight level gliding.
*V_s_*(*H_A_*) (knots)	UAV sink rate at *H_A_*
$V_{sw}\left(H_{A}\right)$ (knots)	Total UAV sink rate at *H_A_*
*V_sφ_* (knots)	UAV sink rate when turning.
$w_{H_{A}}$ (knots)	Downward wind forecast component at *H_A_*
*W* (lbf)	UAV weight at the contingency point, CP
Δ*h* (ft)	Altitude bin height used for estimating altitude drop
Δ*H* (ft)	UAV altitude drop after a travel time of *T_i_*
*ρ* (slug/ft^3^)	Air density value at UAV’s altitude
*φ* (rad)	Bank angle of the turn
*ω* (rad/s)	Turn rate
